# High-intensity interval training alleviates exhaustive exercise-induced HSP70-assisted selective autophagy in skeletal muscle

**DOI:** 10.1186/s12576-023-00884-2

**Published:** 2023-11-21

**Authors:** Jiao Lu, Liu-mei Zhang, Jing-jing Liu, Yu-ting Liu, Xiao-ye Lin, Xue-qi Wang, Yuan Zhang, Qiang Tang, Lin Liu

**Affiliations:** 1 School of Sports and Health, Nanjing Sport Institute, Nanjing, 210014 China; 2 Jiangsu Collaborative Innovation Center for Sport and Health Project, Nanjing, 210014 China; 3 School of Sports and Health, Shanghai University·of Sport, Shanghai, 200000 China

**Keywords:** Autophagy, Chaperone, Exhaustive exercise, High-intensity interval exercise, Skeletal muscle

## Abstract

This study was designed to probe the effect of chaperone-assisted selective autophagy (CASA) on the maintenance of proteostasis during exhaustive exercise and uncover the alteration of CASA in muscle fibers with pre-high-intensity interval training (HIIT) intervention-induced muscle adaptation in response to exhaustive exercise. Rats were randomly divided into a control group; an exhaustive exercise group; and an HIIT + exhaustive exercise group. Results show myofibril damage and BiP levels were increased after exhaustive exercise, and the levels of the HSP70, BAG3, ubiquitin, autophagy-related proteins, and their interactions were increased. HIIT intervention before exhaustive exercise could decrease myofibril injury and BiP levels, accompanied by down-regulation of HSP70/BAG3 complex and selective autophagy. In conclusion, exhaustive exercise promotes CASA to clear protein aggregation for keeping proteostasis in muscle fibers; pre-HIIT intervention improves myofibril injury and unfold protein response caused by exhaustive exercise, which might contribute to inhibit the augmentation of CASA.

**Supplementary Information:**

The online version contains supplementary material available at 10.1186/s12576-023-00884-2.

## Background

Muscle fibers demand strengthened proteostasis mechanisms to maintain their normal structure and function due to their constant subjection to mechanical, heat, and oxidative stresses during contraction. Proteostasis refers to the dynamic balance of complex processes, such as molecular synthesis, structural and functional maturation, folding modification, localization and transport, functional recovery, and degradation of proteins in cells [1]. A major challenge in proteostasis is to prevent the harmful consequences of unfolded, misfolded, or damaged proteins that severely interfere with cell function [2]. According, autophagy serves as a protein disposal system and is upregulated for the adaptation of radical contraction in muscle fibers during majority exercise models [3]. In most low- and medium-exercise models, autophagy increases gently and appears non-selective to sustain the energy supply and maintain proteostasis, which might be one of the important mechanisms of exercise-induced skeletal muscle adaptation [4, 5]. However, in prolonged, strenuous, and eccentric exercise, with the increase in muscle damage and unfolding protein response, proteasome system would be overloaded leading to accumulation of these proteins. So that autophagy might arise for producing more defined degradation targets [6, 7]. Under this circumstance, p62 is recruited to bind accumulated substrates and degrade them through selective autophagy [8]. The upstream mechanism to regulate this process might be involved the mediation of HSP70, an important molecular chaperone that could provide specificity for substrate selection to autophagy pathway in response to stress, which was also called chaperone-assisted selective autophagy (CASA) [9]. Considering that regular exercise training could enable muscle fibers to acquire the adaptation of elevated non-selective autophagy, it is of interest that, given the hypothesis, CASA may be recruited for the degradation of accumulated substrates under prolonged, strenuous, and eccentric exercise models, whether this mechanism would be relived in muscle fiber after exercise-induced adaptation.

High-intensity interval training (HIIT) defined as short bursts of vigorous exercise, interspersed with periodic intervals of rest or low-intensity exercise, which could be viewed as “stress phases” and “recovery phases” for skeletal muscle fibers [10]. Different from other exercise models, muscle fiber suffers from energy deprivation, relative ischemia, and hypoxia and oxidative stress in the stress phase during HIIT, but these negative conditions are reversed in the recovery phase before muscle injury occurs, which causes muscle to adapt to higher intensity exercise [11, 12]. Among plenty of exercise models, HIIT was recommended as the best way to invoke muscle adaptation within a short time period, to efficiently achieve muscle improvement [13]. Therefore, the present study clarified the expression of p62-mediated selective autophagy in response to clearance of damaged, unfolded/misfolded proteins induced by exhaustive exercise through the assistance of molecular chaperone HSP70. Additionally, we used HIIT as an effective method to acquire muscle adaptation against exhaustive exercise-induced muscle injury to explore alterations in CASA in muscle fibers.

## Materials and methods

### Animals and experimental protocol

In this research, 45 male Sprague–Dawley rats (8 weeks of age) were obtained from Shanghai SLAC Laboratory Animal Co., Ltd. (Shanghai, China) and divided into three equal groups (*n* = 15/group), including a control group (Group C), an exhaustive exercise group (Group EE), and an HIIT + exhaustive exercise group (Group HIIT + EE). The rats were allowed free access to standard chow and water and were housed in a temperature-controlled room under a 12 h light/dark cycle. All of the animal care and experimental procedures were approved by the Ethics Committee for Science Research of the Nanjing Sport Institute (approval number: GZRDW-2020–02).

Before formal experimental training, treadmill habituation was carried out for three consecutive days at 15 m/min and 0% grade for 10–20 min each day. Afterward, all animals were allowed 1 day of rest. Rats in Group C were placed on non-moving treadmills to normalize handling stress. In Group EE, rats ran on treadmills at 25 m/min and 0% grade until exhaustion, and we then assessed whether they were able to orient themselves upright when placed on their back. In Group HIIT + EE, rats ran on treadmills at 28 m/min and 0% grade for four periods of 10 min interspersed with 10 min of rest each for the HIIT model, so that they approached 80% VO2max, according to a previous study [14]. This protocol was implemented for three consecutive days; then, 24 h later, exhaustive exercise was encouraged. All rats in the exercise groups were allowed a 5 min warmup period before exercise, and the rats in Group HIIT + EE participated in a 5 min cool-down training period at the end of the HIIT model. The time and distance of the rats during exhaustive exercise were recorded. The standard of exhaustion in rats is that the abdomen is close to the ground, the limbs are limp, and the righting reflex is temporarily disappeared. Animal sampling was completed within 30 min.

### Material collection

After the exercise, all rats were anesthetized with 5% pentobarbital sodium (30 mg/kg) immediately. Blood was collected from the inferior vena cava and centrifuged at 1000 r/min for 10 min, and plasma was taken and stored in a − 80 °C refrigerator. Seven rats per group were selected for perfusion fixation; thereafter, gastrocnemius tissues were excised and fixed in 4% paraformaldehyde for histology analysis. The other eight rats’ gastrocnemius tissues were rapidly excised and stored at − 80 °C for western blot analysis.

### Transmission electron microscopy (TEM)

After perfusion and fixation, gastrocnemius muscle samples were cut into 1 × 1 × 1 mm^3^ tissue blocks, and skeletal muscle samples were fixed in 2.5% glutaraldehyde in phosphate-buffered saline and fixed in 1% osmic acid for 1 h, and then processed using the conventional techniques. Ultrathin sections (70 nm) were stained with lead citrate and uranyl acetate and inspected by TEM (JEM-1400; Jeol, Tokyo, Japan). Five horizons were taken in each sample; therefore, total 15 horizons per group were taken for Z-disk integrity analysis by Image-Pro Plus 6.0 (Media Cybernetics, Rockville, MD, USA). The Z-disk integrity density in myofiber was calculated to display the myofibril injury and indicate filamin disintegration.

### Western blotting

Total protein was extracted from gastrocnemius tissue, and the protein levels in these extracts were measured via bicinchoninic acid protein assay (Epizyme, Shanghai, China). Proteins samples were subjected to sodium dodecyl sulfate-polyacrylamide gel electrophoresis on 10–12.5% separation gels and transferred to PVDF membranes, which were blocked with 5% milk (dissolved in TBST) for 1 h. All PVDF membranes were incubated overnight at 4 °C with the following specific primary antibodies: Beclin 1 (11,306-1-AP, 1:1000; Proteintech Group, Rosemont, IL, USA), p62 (sc-48402, 1:100; Santa Cruz Biotechnology, Dallas, TX, USA), LC3 (14,600-1-AP, 1:1000; Proteintech), ubiquitin (ab140601, 1:3,000; Abcam, Cambridge, UK), BiP (BS6479, 1:1000; BioWorld, Irving, TX, USA), HSP70 (4872 s, 1:1000; CST, Danvers, MA, USA), BAG 3(68,076-1-Ig, 1:5000; Proteintech Group), and GAPDH (10,494–1-AP, 1:30,000; Proteintech Group). The following day, the PVDF membranes were washed three times with TBST (10 min each time) and incubated in HRP-conjugated secondary antibody for 1 h at 25 °C (room temperature) using either goat anti-rabbit antibody (BS13278, 1:10,000; BioWorld, USA) or goat anti-mouse antibody (7076 s, 1:2,000, CST, USA). A quantitative analysis of proteins was performed using the Image Lab software (Bio-Rad Laboratories, Hercules, CA, USA). GAPDH was used as a loading control to correct the values, and the differences were displayed with the relative value, which was normalized to the control.

### Immunofluorescence

Three wax samples were randomly selected and cut into 4 μm slices. After dewaxing and dehydration, sections were immersed in sodium citrate buffer at 95 °C for 10 min for antigen retrieval. For double-labeling immunofluorescence, sections were blocked with 10% goat serum for 1 h, and then incubated under 4 °C for the night with mixed primary antibodies, specifically rabbit anti-LC3 (diluted at 1:100) and mouse anti–LAMP-2 (diluted at 1:100), rabbit anti-LC3 (diluted at 1:100) and mouse anti-p62 (diluted at 1:100), rabbit anti-ubiquitin (diluted at 1:100) and mouse anti-p62 (diluted at 1:100), and rabbit anti-HSP70 (diluted at 1:100) and mouse anti-BAG3 (diluted at 1:100, 68,076-1-AP; Proteintech). After rinses with phosphate-buffered saline, secondary antibodies were used, including goat anti-rabbit Alexa Fluor 488 (ab150116, 1:500; Abcam) and goat anti-mouse Alexa Fluor 594 (ab150077, 1:500; Abcam). Cell nuclei were stained for 5 min with DAPI. Images were captured with a microscope (400×). Five non-continuous fields were randomly selected from each section, and a total of 15 horizons from each group participated in the statistics. The positive areas and colocalization dots were analyzed with ImageJ (U.S. National Institutes of Health, Bethesda, MD, USA).

### Immunohistochemistry

After dewaxing and dehydration, sections were blocked with 3% H2O2 for 15 min, and then immersed in sodium citrate repair solution at 95 °C for 15 min. After cooling them to room temperature, the slices were blocked with 5% BSA for 1 h, and then incubated at 4 °C for the night with a primary antibody specific to BiP diluted at 1:100. An HRP-labeled secondary antibody (SV-0001; Boster) was used at 37 °C for 30 min. Diaminobenzidine was used to develop color, and counterstaining was performed using hematoxylin. Finally, the slides were observed under a microscope (400×). A total of 15 horizons from each group were taken for morphometric analysis by Image-Pro Plus 6.0 (Media Cybernetics, Rockville, MD, USA). The BiP density in gastrocnemius tissue was calculated to display the degree of positive events.

### Statistical analysis

ImageJ was used for image analysis. All experimental data were processed with GraphPad Prism 8 (GraphPad Software, San Diego, CA, USA), and the results were expressed as mean ± standard error of the mean values. One-way analysis of variance was used to compare the results between groups. *P* < 0.05 was considered statistically significant.

## Results

Exhaustive exercise-induced vigorous autophagosome formulation was crippled by pre-HIIT intervention.

**Fig. 1:**
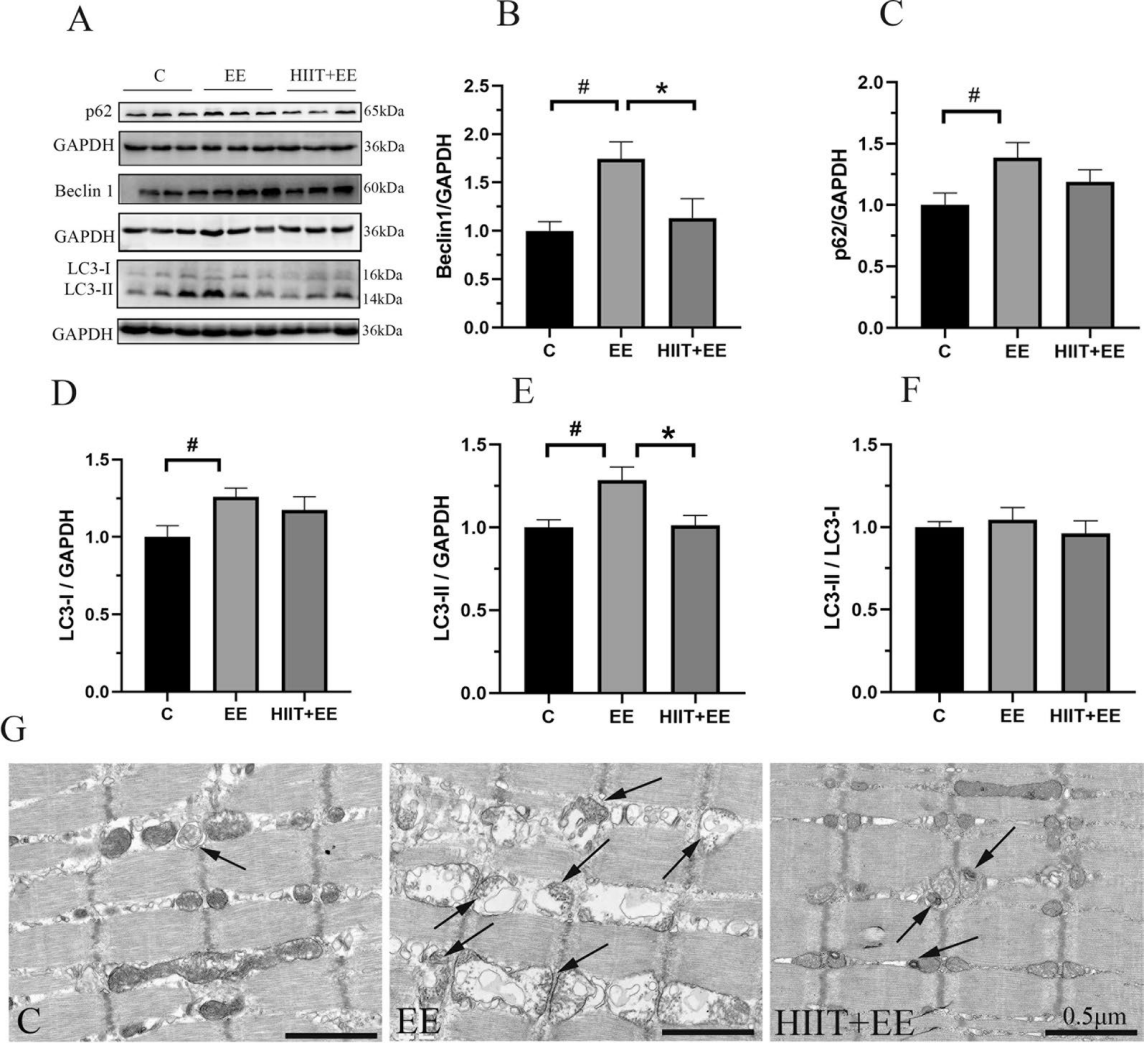
Alterations of autophagosomes in gastrocnemius. **A** Representative blots images of autophagy-related proteins Beclin 1, p62, LC3, and GAPDH in gastrocnemius muscle tissue. **B**–**F** Qualitative analysis of autophagy-related proteins (*n* = 8); **B** protein levels of Beclin 1, **C** protein levels of p62, (**D**, **E**) protein levels of LC3-1 and LC3-II, and (**F**) the ratio of LC3-II/LC3-I. **G** TEM images of autophagic changes in muscle fibers; black arrows show autophagic changes. Scale bars = 0.5 μm. ^#^*P* < 0.05 vs. Group C, ^*^P < 0.05 vs. Group EE

We first examined the levels of autophagy-related proteins, such as Beclin 1, p62, and LC3 in gastrocnemius muscle tissue (Fig. 1A). These proteins play an important role in autophagosome formation and are used wildly in autophagy-level tests. Beclin 1 is recruited during the initiation of autophagy and facilitates the extension of the autophagy membrane [15]. As expected, Beclin 1 levels were elevated significantly after exhaustive exercise (*P* < 0.05) but were crippled by pre-HIIT intervention (*P* < 0.05) (Fig. 1B). p62 served as “cargo,” pulling the substrates into autophagy by interaction with the LC3-II located in the inter-autophagy membrane [16]. p62 is digested with substrates in autolysosomes and declines when non-selective autophagy becomes active. In this work, an accumulation of p62 was observed after exhaustive exercise (*P* < 0.05) and persisted at a high level even with pre-HIIT intervention (*P* > 0.05) (Fig. 1C). As LC3-II aggregates both inside and outside the autophagy membrane, the expression of LC3-II may be used for quantitative analysis of autophagosomes via both western blot (Fig. 1D–F). Both LC3-I and LC3-II levels were increased significantly after exhaustive exercise (*P* < 0.05). With pre-HIIT intervention, the LC3-II level decreased (*P* < 0.05). Finally, there were no significant differences in LC3-II/ LC3-I among the three groups. The assessment of autophagosome morphological characteristics was carried out via TEM imaging of muscle fibers, and the TEM results of gastrocnemius muscle tissue from Group EE demonstrated that numerous autophagosomes appear among myofibrils; meanwhile, the number of autophagosomes was decreased in the pre-HIIT model (Fig. 1G).

The degradation of autophagosomes was consistent with their formulation in both exhaustive exercise and pre-HIIT intervention.

**Fig. 2:**
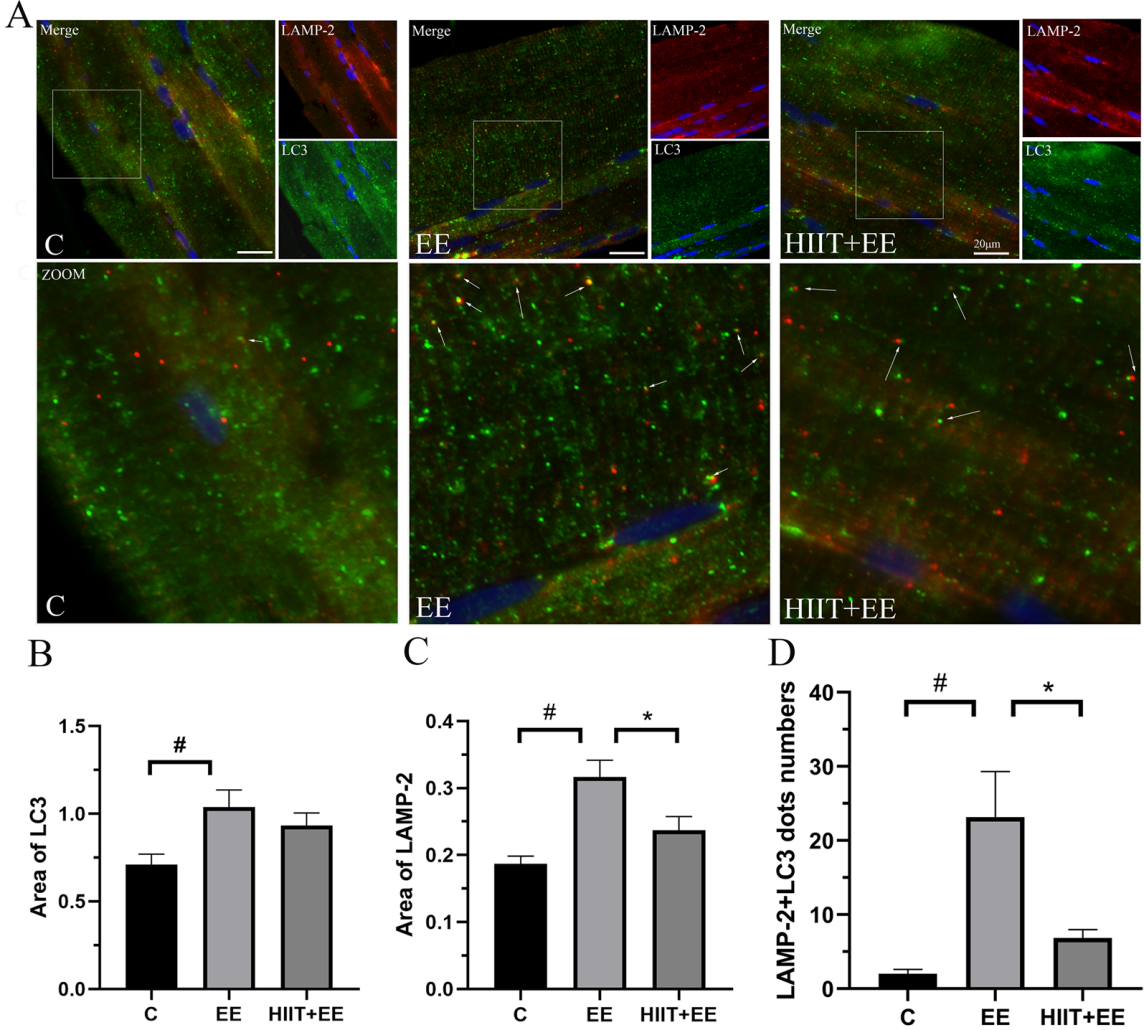
Assessment of the degradation of autophagosomes in gastrocnemius muscle tissue. **A** Immunofluorescence staining of autophagosome marker LC3 (green) and lysosome marker LAMP-2 (red). Colocalization of LC3 and LAMP-2 is demonstrated using yellow dots indicating autolysosomes; white arrows denote autolysosomes. Scale bars = 20 μm. **B**, **C** Analysis of immunoreactive areas of LC3 and LAMP-2. **D** Statistical analysis of LC3 and LAMP-2 colocalization dots (*n* = 15). ^#^*P* < 0.05 vs. Group C, ^*^P < 0.05 vs. Group EE

To evaluate the degradation of autophagosomes, we next assessed the degradation of autophagosomes by immunofluorescent labeling of LC3 and LAMP-2. LC3 is located in the autophagosome membrane, while LAMP-2 is located in the lysosome membrane, and these are often used to label autophagosomes and lysosomes by immunofluorescence, respectively. Consequently, the colocalization areas of LC3 and LAMP-2 identify autolysosomes. The colocalization areas demonstrated rough dots brightly and clearly, so that it was easy to count them for statistics, which was recommended in “Guidelines for the use and interpretation of assays for monitoring autophagy” [17]. The analysis of subsequent double-labeling immunofluorescence was carried out in the same way. In this experiment, LC3 demonstrated bright green puncta, while LAMP-2 exhibited red puncta, and their colocalization area demonstrated yellow fluorescence (Fig. 2A). Compared to Group C (Fig. 2B, C), elevated LC3 and LAMP-2 immunoreactivity areas were observed in Group EE to a significant difference (*P* < 0.05), while LAMP-2 immunoreactivity areas in Group HIIT + EE were decreased significantly compared to those in Group EE (*P* < 0.05). The colocalization dots of LC3 and LAMP-2 immunofluorescence showed that muscle fibers in Group EE demonstrated a significant increase in autolysosomes compared to those in Group C (*P* < 0.05), while HIIT pre-intervention alleviated the high level of autolysosomes induced by exhaustive exercise (*P* < 0.05) (Fig. 2D). Additionally, we examined levels of cathepsin D, a digestive enzyme of lysosomes, which is often used to estimate lysosomal digestion. The detection results are consistent with the above results (Additional file 1: Fig S1, which demonstrates the expression level of cathepsin D). Based on the results of autophagic formation in Group HIIT + EE, elevated autophagy flux during exhaustive exercise was alleviated by HIIT pre-intervention p62-mediated selective autophagy promoted the degradation of ubiquitin proteins during exhaustive exercise.

**Fig. 3:**
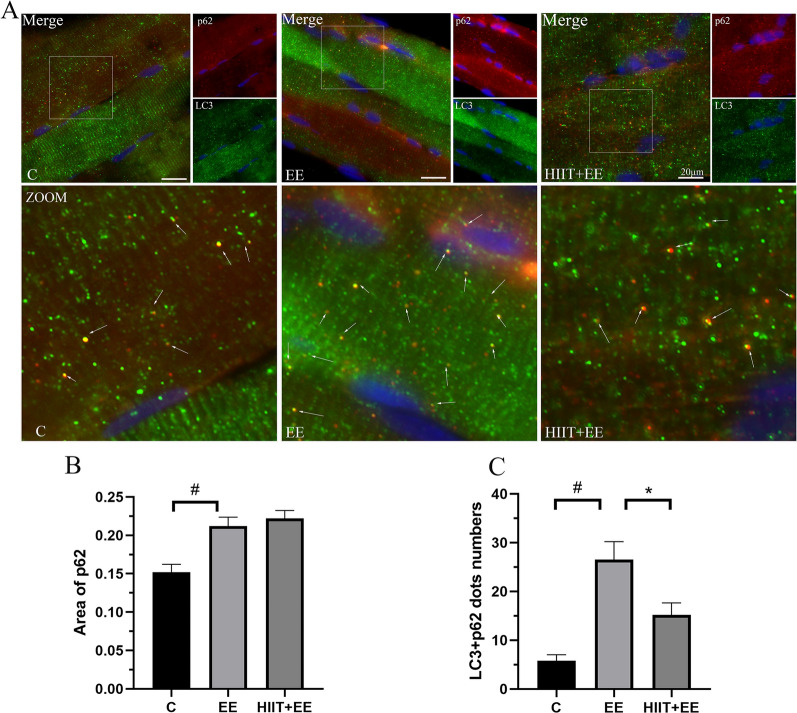
Autophagic cargo p62 associated with the autophagosome marker LC3-1n muscle fibers. **A** Immunofluorescence staining of the autophagosome marker LC3 (green) and autophagic cargo p62 (red). Colocalization of LC3 and p62 is demonstrated with yellow dots indicating autolysosome association of LC3 and p62; white arrows denote the colocalization of LC3 and p62. Scale bars = 20 μm. **B** Analysis of the immunoreactive area of p62. **C** Statistical analysis of LC3 and p62 colocalization dots (*n* = 15). ^#^*P* < 0.05 vs. Group C, ^*^P < 0.05 vs. Group EE

To verify the hypothesis that the accumulation of p62 promotes selective autophagy in muscle fibers during exhaustive exercise, we assessed p62-mediated selective autophagy through LC3 and p62 immunofluorescent labeling. LC3-II showed bright green immune-positive spots, while p62 exhibited red puncta, and colocalization demonstrated yellow puncta, indicating the existence of autophagy complexes carrying degraded waste (Fig. 3A). Quantitative analysis of p62 immunoreactivity areas and their colocalization dots was performed, and the results showed that p62 immunoreactive areas in Group EE were larger than those in Group C (*P* < 0.05) (Fig. 3B). The colocalization dots also showed that there were many fully loaded autophagy complexes within muscle cells in Group EE, and more were present in this group than in Group C (*P* < 0.05). However, these phenomena were significantly reduced by HIIT training (Fig. 3C), as the expression of colocalization dots of LC3 and p62 was decreased significantly in Group HIIT + EE compared to in Group EE (*P* < 0.05).

**Fig. 4:**
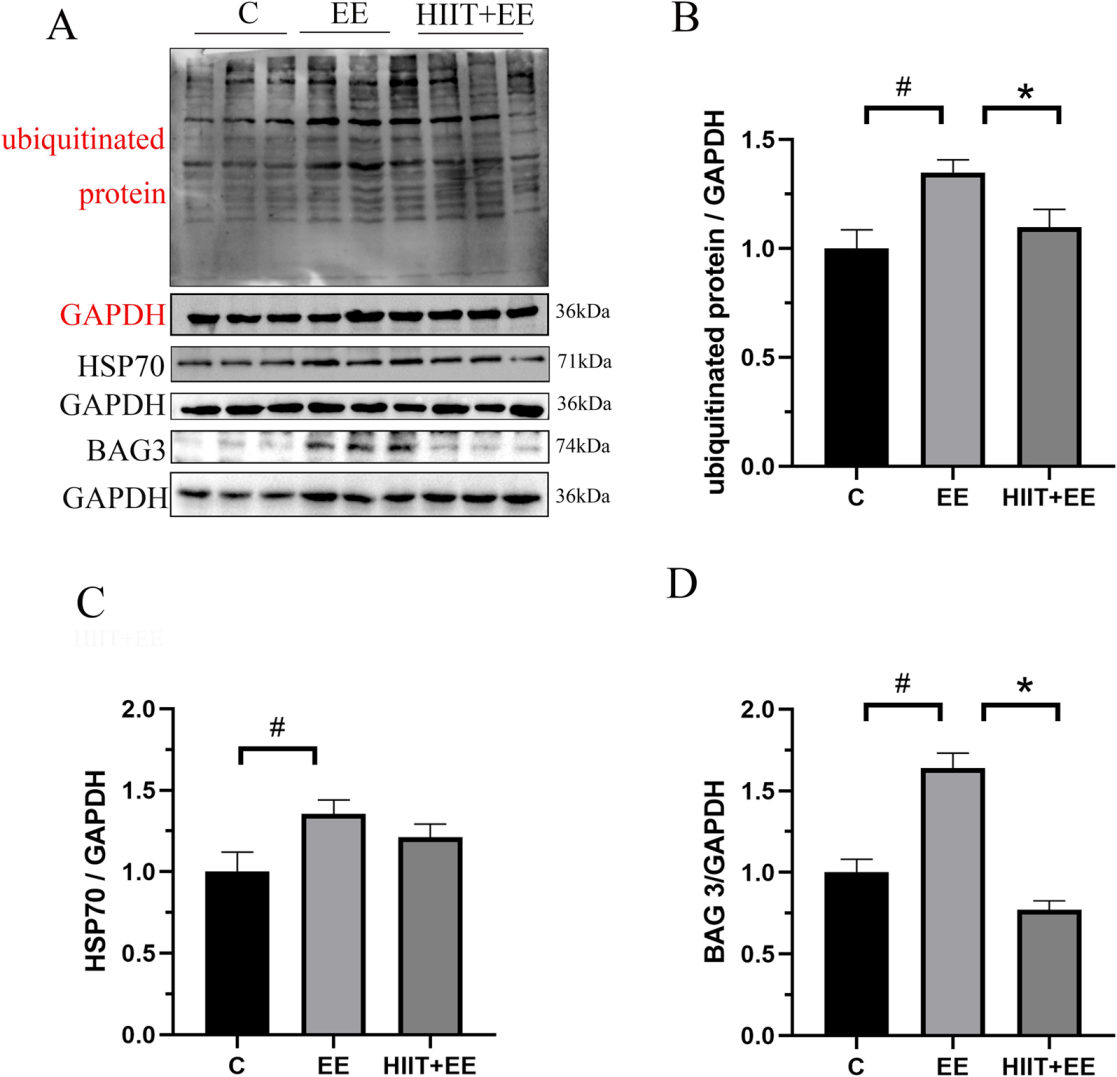
The expression of HSP70 complex-related proteins. **A** Blot image of ubiquitinated protein, HSP70 and BAG3. **B**–**D** Qualitative analysis of ubiquitin, HSP70 and BAG3 (*n* = 8)

**Fig. 5:**
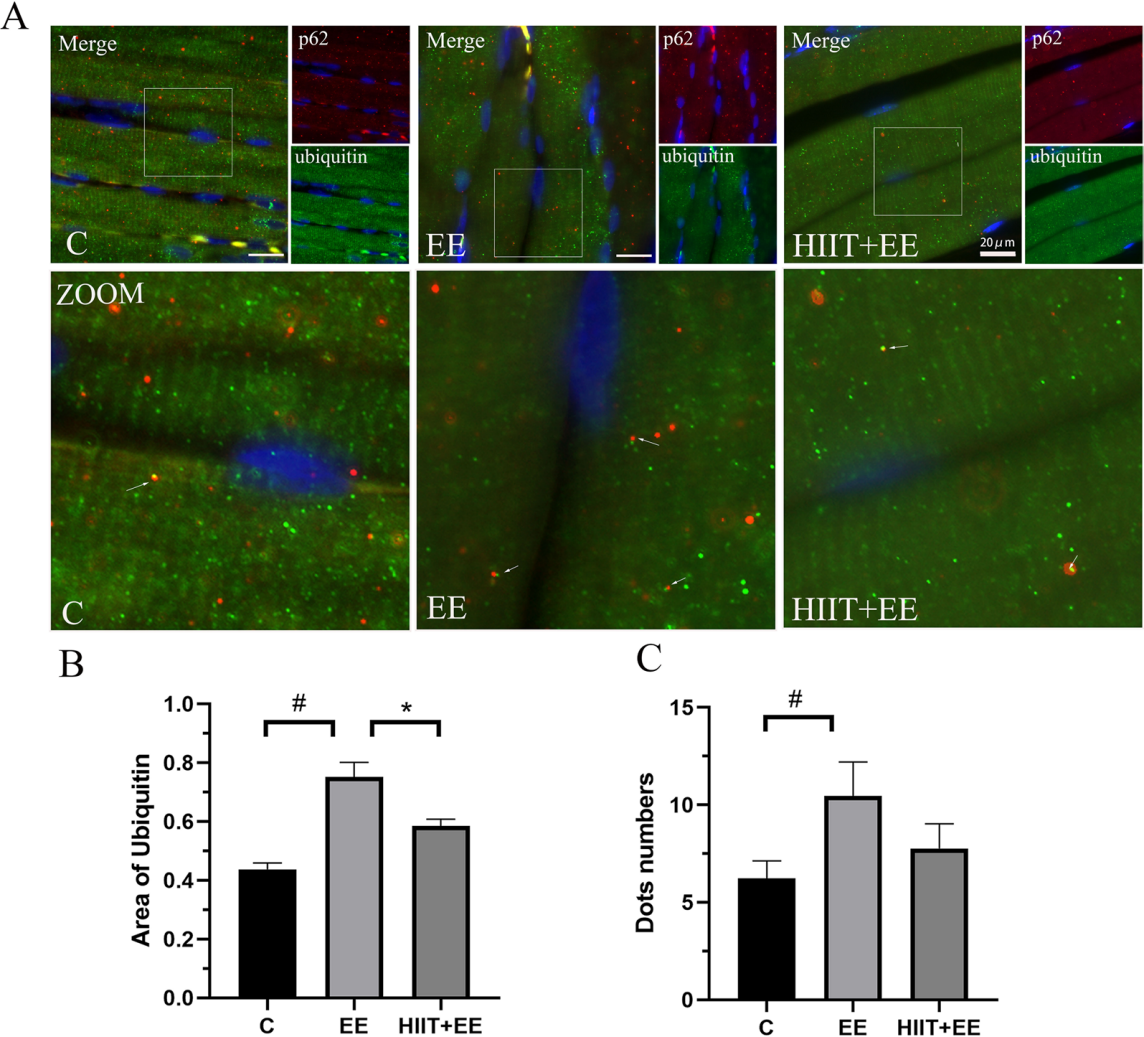
Ubiquitinated substrates were recognized by p62 in muscle fibers. **A** Immunofluorescence staining of ubiquitin (green) and autophagic cargo p62 (red). Colocalization of ubiquitin and p62 is demonstrated using a yellow area indicating the association of ubiquitin and p62; white arrows denote the colocalization of ubiquitin and p62 dots. Scale bars = 20 μm. **B** Analysis of the immunoreactive area of ubiquitin. **C** Statistical analysis of ubiquitin and p62 colocalization dots (*n* = 15). ^#^*P* < 0.05 vs. Group C, ^*^P < 0.05 vs. Group EE

Ubiquitinated proteins are important candidates of substrates that are recognized by p62 and conducted to autophagosomes for degradation, which serves as a compensatory mechanism for the ubiquitin–proteasome proteolytic system when unfolded/misfolded proteins are overloaded and aggregated. To assess the degradation of ubiquitinated proteins via selective autophagy, the ubiquitinated protein level was measured and double immunofluorescent labeling of ubiquitin and p62 was performed in this work (Figs. 4A, 5). The ubiquitinated proteins levels were increased significantly after exhaustive exercise (*P* < 0.05), but this increase was also crippled by pre-HIIT intervention (*P* < 0.05) (Fig. 4B). The immunofluorescence results showed that ubiquitinated proteins demonstrated a bright green fluorescence signal, while p62 showed a red signal, and colocalization demonstrated a yellow signal, indicating the combination of ubiquitinated proteins and p62 (Fig. 5A). The quantitative results of ubiquitin immunoreactivity areas were similar to the levels of ubiquitinated proteins in western blotting (*P* < 0.05). The colocalization dots of ubiquitin and p62 were consistent with the level of selective autophagy, and elevated colocalization dots (*P* < 0.05) were decreased significantly by pre-HIIT intervention (*P* < 0.05) (Fig. 5B, C). Up-regulated HSP70s were recruited to binding damaged, unfolded/misfolded proteins as ubiquitinated clients via BAG3.

**Fig. 6:**
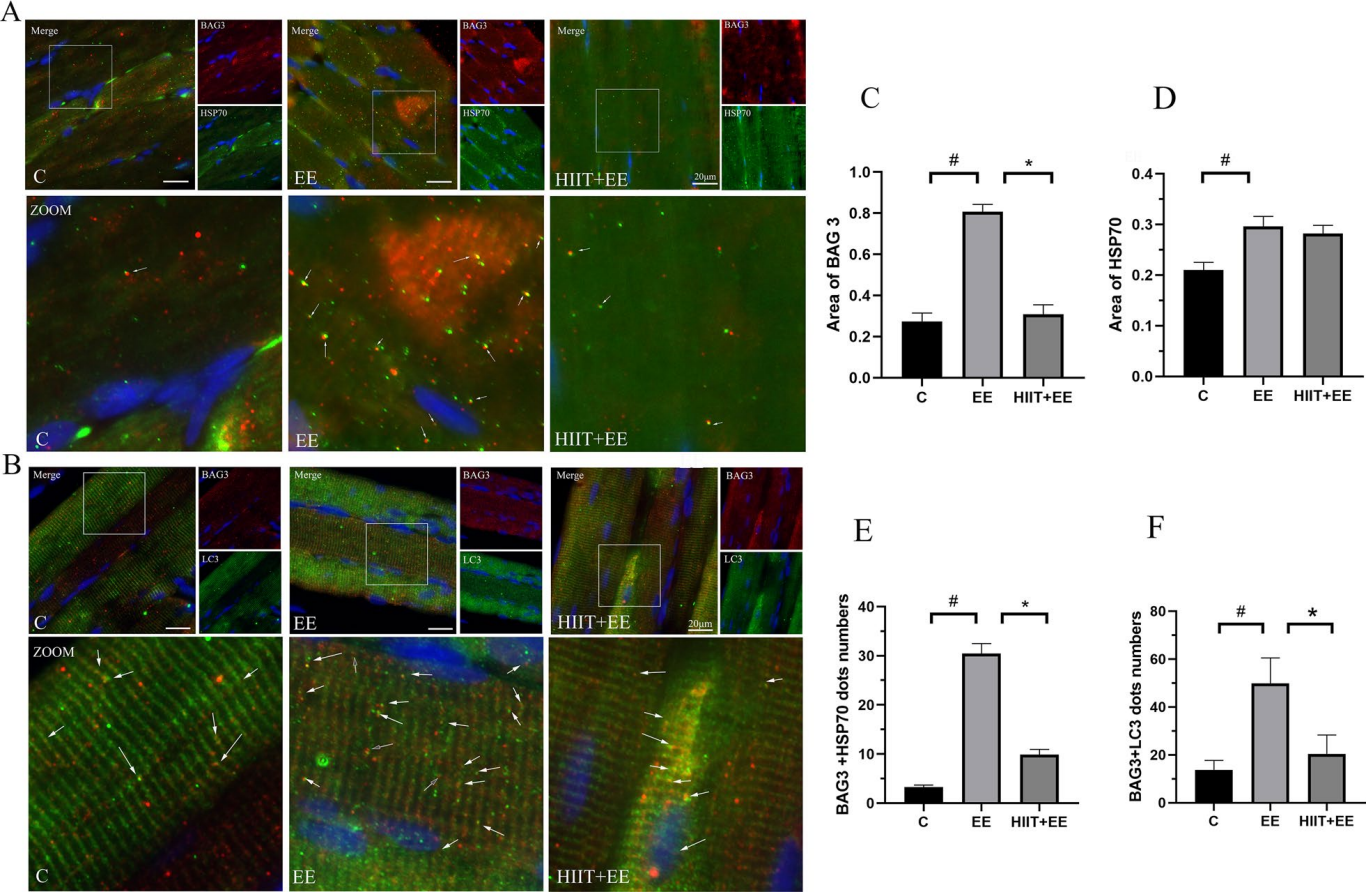
BAG3 specifically directs HSP70 to form a complex for degradation through the autophagy pathway. **A** Immunofluorescence staining of chaperone HSP70 (green) and cochaperone BAG3 (red). Colocalization of HSP70 and BAG3 is demonstrated as a yellow area indicating the association of HSP70 and BAG3; white arrows denote the colocalization of HSP70 and BAG3. Scale bars = 20 μm. **B** Immunofluorescence staining of chaperone LC3 (green) and cochaperone BAG3 (red). Colocalization of LC3 and BAG3 is demonstrated as a yellow area indicating the association of LC3 and BAG3; white arrows denote the colocalization of LC3 and BAG3. Scale bars = 20 μm. **C**, **D** Analysis of the immunoreactive areas of BAG3 and HSP70. **E** Statistical analysis of HSP70 and BAG3 colocalization dots (*n* = 15). **F** Statistical analysis of LC3 and BAG3 colocalization dots (*n* = 15). ^#^*P* < 0.05 vs. Group C, ^*^P < 0.05 vs. Group EE

To further investigate the degradation of damaged, unfolded/misfolded proteins via HSP 70-assisted selective autophagy, we first assessed the expression of HSP70, BAG3 and their interaction (Figs. 4A, 6). The western blotting results showed that the HSP70 was upregulated following exhaustive exercise (*P* < 0.05). However, pre-HIIT intervention failed to downregulate the level of HSP70 (*P* > 0.05) (Fig. 4C). Exhaustive exercise significantly increased the expression of BAG3 (*P* < 0.05), but decreased after pre-HIIT intervention (*P* < 0.05) (Fig. 4D). Then, the interaction between HSP70, BAG3, and LC3 was also evaluated by double-labeling immunofluorescence (Fig. 6A, B). The results demonstrated that HSP70 showed a bright green fluorescence signal, while BAG3 showed a red signal, and colocalization demonstrated a yellow signal, indicating the interaction of HSP70 and BAG3. The quantitative results of HSP70 and BAG3 areas were similar to the levels of those found in western blotting (Fig. 6C, D). The colocalization dots of HSP70 and BAG3 were also consistent with the level of selective autophagy, in that colocalization dots were increased after exhaustive exercise (*P* < 0.05) but decreased significantly by pre-HIIT intervention (Fig. 6E) (*P* < 0.05). To confirm that exhaustive exercise-induced unfolded/misfolded proteins are degraded by HSP70-mediated CASA. We detected the colocalization of chaperone complex marker protein BAG3 and autophagy marker protein LC3. LC3 was showed a bright green fluorescence signal, BAG 3 showed a red signal, and colocalization demonstrated a yellow signal indicating the colocalization of LC3 and BAG 3. The results displayed the colocalization dots of BAG 3 and LC3 which were increased after exhaustive exercise (*P* < 0.05) but decreased significantly by pre-HIIT intervention (*P* < 0.05) (Fig. 6F).

**Fig. 7:**
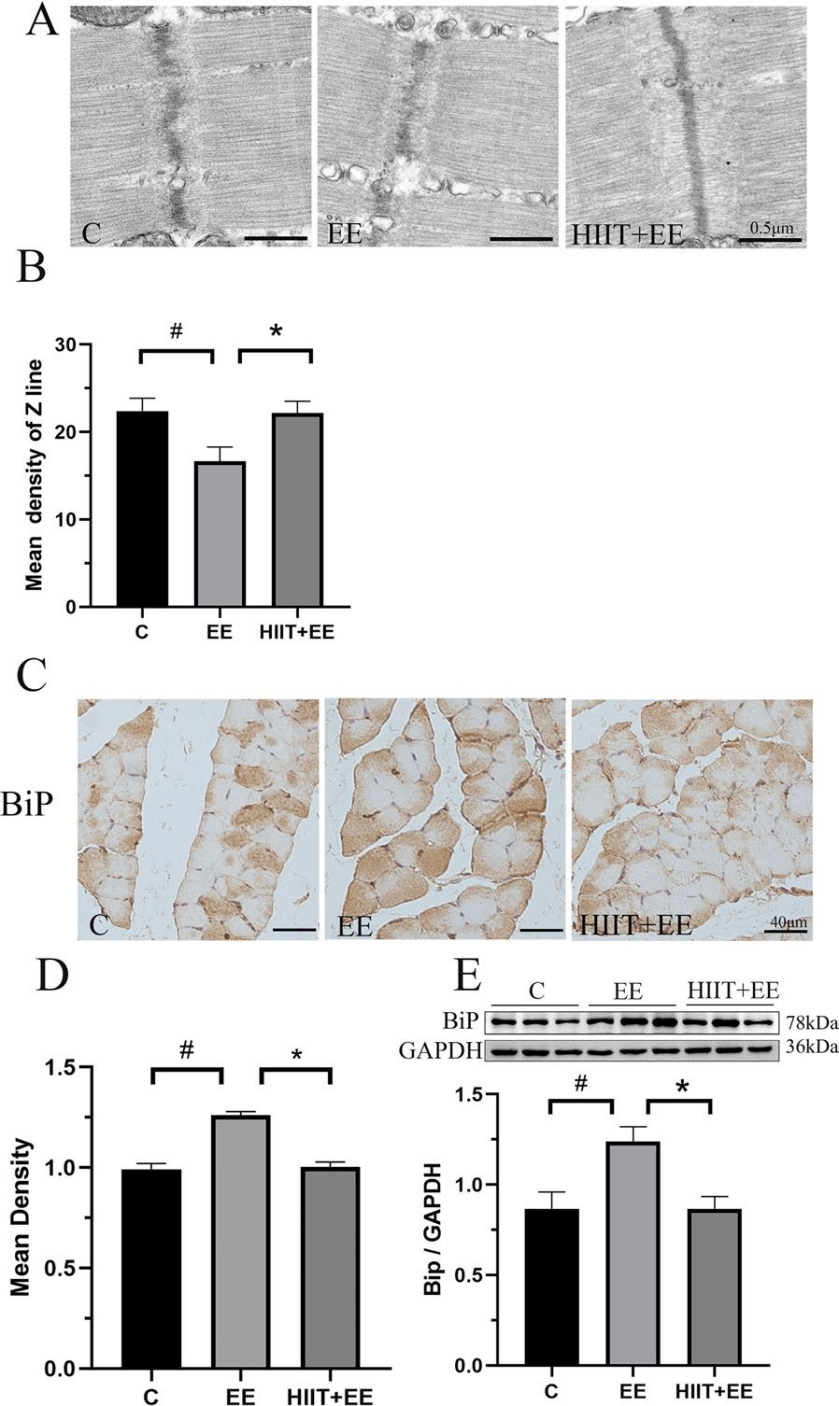
Assessment of Z-disk damage and UPR-related protein levels. **A** TEM images of Z-disk in myofibrils, Scale bars = 0.5 μm. **B** Qualitative analysis of Z-disk density (*n* = 15). **C** Immunohistochemistry of BiP in gastrocnemius muscle tissue, Scale bars = 40 μm. **D** Qualitative analysis of BiP density (*n* = 15). **E** Blot images and qualitative analysis of BiP (*n* = 8). ^#^*P* < 0.05 vs. Group C, ^*^P < 0.05 vs. Group EE

Second, we examined the Z-disk density as a means to indicate myofibril injury and filamin disintegration, which previous work has examined (Fig. 7) [18]. Z-disk alterations are commonly observed in myofibril injury and often accompanied by the disintegration of the structural component, filamin, which is terminated by CASA. The result revealed that Z-disk streaming and disorganization were observed in Group EE, and the Z-disk density was decreased significantly in this group compared to that in Group C (*P* < 0.05) (Fig. 7A, B). Furthermore, with HIIT intervention, the structure of Z-disk was improved and the density of Z-disk was increased significantly (*P* < 0.05). Then, to verify that ubiquitinated proteins were attributable to the exhaustive exercise promoting UPR-induced overexpression of unfolded/misfolded proteins, we assessed the ER chaperone protein BiP, a central regulator of ER homeostasis, by western blotting and immunohistochemistry (Fig. 7C, D and F). Immunohistochemistry results revealed strong BiP expression in gastrocnemius tissue following exhaustive exercise. The analysis of the BiP-positive area further confirmed that BiP expression was significantly increased after exhaustive exercise (*P* < 0.05), whereas the upregulated BiP expression was reduced significantly with pre-HIIT intervention (*P* < 0.05). Western blot assay also demonstrated that the expression of BiP was significantly increased in Group EE compared to Group C (*P* < 0.05) and was decreased in Group HIIT + EE (*P* < 0.05).

## Discussion

Autophagy can be activated by exercise and plays different roles according to the intensity, amount, and mode of physical activity [19–21]. Previous studies have reported that, in skeletal muscle fibers, autophagy is recruited to serve as a compensatory mechanism for sustaining energy demands and nutrient supply during exercise-induced energy crisis, a phenomenon which is probably regulated by the BCL-2-mediated autophagy pathway for glucose homeostasis [22]. Additionally, autophagy also plays an important role in the degradation of organelles or proteins to retain dynamic proteostasis for the maintenance of muscle ultrastructure and function [23, 24]. However, in prolonged, strenuous, and eccentric exercise models, the upregulation of autophagy-related proteins is always accompanied by the elevated expression of p62, which should be digested with substrates and declined when non-selective autophagy becomes active [25]. Blockage of autophagosome degradation accounts for the accumulation of p62 and contributes to the muscle damage induced by exercise. Because the disorder of autophagosome degradation can also cause the accumulation of toxic substances in the cytoplasm, which is an important factor in cell damage and apoptosis [26]. However, more opinions tend to support the idea that the elevation of p62 serves as an ubiquitin receptor to mediate the degradation of exercise-induced damaged substrates through selective autophagy. Many works have shown that elevated p62 is translocated to the damaged mitochondria and induces mitophagy, which might connect to the survival and maintenance of persistently normal mitochondrial [27–29]. In our study, we observed that both autophagosomes and autolysosomes were increased, indicating that no disorder of autophagy degradation occurs during exhaustive exercise. Furthermore, the elevated p62 was recruited around to the LC3, bunding more ubiquitinated substrates with autophagosomes. These phenomena unveiled that the accumulation of p62 was summoned for selective autophagy, and substrate degradation rather than energy supply is preferable for increased autophagy during exhaustive exercise.

Interestingly, rats under pre-HIIT intervention showed reduced autophagy levels, even with prolonged time and distance of exhaustive exercise. Moreover, muscle adaptation was acquired after HIIT, leading to reduced damage of organelles or proteins that might be important components of autophagic degradation during exhaustive exercise. Consequently, the metabolic products such as damaged organelles or proteins produced by exercise in skeletal muscle might determine the expression of selective autophagy during exercise.

In fact, basic selective autophagy is essential for the degradation of damaged organelles and proteins in myocyte fibers to retain proteostasis and promote the repair process [30]. During normal muscle contraction, mechanical damage of proteins around the Z-disk is specifically recognized by the chaperone complex that composes the molecular chaperone HSP70 and cochaperone BAG3 [31, 32]. Then, the complex undergoes ubiquitination with the assistance of CHIP (which acts as an E3–ubiquitin ligase) and is recognized by p62 to degrade through selective autophagy. Apparently, it is inevitable that great myofibril damage occurs because of too great a mechanical load and contraction under excessive exercise, which is evidenced by the observation of Z-disk streaming and disorganization in myofibrils and the elevated serum CK levels present after exhaustive exercise (see Additional file 1: Fig S2, which demonstrates the content of skeletal muscle injury) [18]. Moreover, the damaged structure of the Z-disk was inconsistent with the increased expression levels of HSP70 and BAG3 and their interaction, indicating that the degradation of Z-disk structure proteins was strengthened during exhaustive exercise. This process is essential for activation and maturation of satellite cells, the mediated regenerative response, maintaining muscle function, and inhibiting apoptosis-caused muscle atrophy [33, 34]. Otherwise, in a BAG3 − / − model, structure proteins comprising Z-disk failed to degrade, leading to early pathological changes in the sarcomere and setting the stage for subsequent muscle apoptosis. Therefore, mice died of muscle atrophy-induced respiratory or heart failure [35, 36]. Even during single resistance exercise, HSP70 and BAG3 complex-assisted selective autophagy demonstrably responded for muscle maintenance and adaptation [18]. Consequently, the upregulation of the HSP70 chaperone complex in response to exercise-induced myofibril damage might promote repair and the inhibition of apoptosis through interactions with selective autophagy.

Apart from the degradation of terminated Z-disk structure proteins, the accumulation of unfolded/misfolded proteins might be another key factor to activate HSP70 chaperone complex-assisted selective autophagy in muscle fibers under stress conditions. These proteins probably arise from prolonged exercise-induced UPR [37]. Recent works have provided important clues that, beyond the refolding ability of ER-located molecular chaperones during the unfolding protein response, BiP could also facilitate protein unfolding or misfolding for degradation through a process referred to as ER-associated degradation, which involves the proteasome and autophagy system [38–40]. In our work, the exorbitant expression of BiP after exhaustive exercise indicates that the chaperone system in the RE lumen was overloaded, leading to the accumulation of unfolded/misfolded proteins in cytoplasm. Subsequently, it is possible that cytoplasmic HSP70s take over tasks to deal with these proteins as part of their molecular chaperone duty. These proteins initiate refolding with the help of HSP70. However, in this study, exhaustive exercise-induced UPR associated with an increase in HSP70 resulted in the augmentation of HSP70 complex and autophagy. These results indicate the exhaustion of HSP70 folding capacity. Then, overloaded unfolded/misfolded proteins began to degrade via different pathways determined by cochaperones interacting with HSP70 [41, 42]. The BAG family proteins are a kind of cochaperone family that potentiates HSP70 client proteins to degrade through proteasome and autophagy, respectively [43]. Both BAG1 and BAG3 proteins could release unfolded/misfolded proteins from HSP70 through the facilitation of ADP/ATP exchange on the HSP70 N-terminal domain. BAG1 could interact with the proteasome through an ubiquitin-like domain and promote HSP70 substrate degradation by the proteasome pathway [44]. This is why, the ubiquitin–proteasome hydrolysis system was activated after prolonged exercise. Moreover, the preloading complex composed of HSP70, BAG3, and ubiquitinated proteins was targeted by p62 and subsequently degraded through the autophagy pathway [45, 46]. Our results confirmed this mechanism, indicating that HSP70-mediated CASA helps to degrade unfolded/misfolded proteins to maintain proteostasis in an exhaustive exercise model.

Both the chaperone system and autophagy represent protein management alternatives for stressed cells. The chaperone system is generally conservative in nature in that it prevents protein aggregation and facilitates protein refolding, whereas autophagy results in the degradation of proteins and organelles. In our work, we discovered that the chaperone system and autophagy cooperate with one another to degrade damaged organelles and UPR caused by exhaustive exercise. However, with the improvement of muscle damage induced by pre-HIIT intervention, the expression of HSP70 and autophagy demonstrated quite different trends, in that selective autophagy was decreased, while HSP70 levels remained high. HSP70 also plays an important role in protein refolding and disaggregation, apart from assisted substrate degradation through the autophagy pathway. It is possible that upregulated HSP70 was prioritized to restore cellular homeostasis by maintaining proper protein structure, and then inhibited the autophagy-induced degradation [47]. This mechanism was declared by Karol’s earlier work, where, with heat-preconditioning or glutamine administration, overexpressed HSP70 prevented starvation-induced autophagy in vitro or exercise-induced autophagy in vivo [3]. Considering the expression of HSP 70 level sill remained high, but HSP 70/BAG3 complex decreased with pre-HIIT intervention, the overexpressed HSP 70 might make contribute to refold exhaustive exercise-induced unfolded/misfolded proteins and inhibit CASA. Moreover, HIIT could bring out protective effects against exhaustive exercise-induced muscle injury, reducing the aggregation of damaged organelle and protein that could be autophagic substrates, relieving the burden of autophagic degradation. Taken together, HIIT intervention before exhaustive exercise improves myofibril injury and the unfold protein response caused by exhaustive exercise, which might help inhibit the augmentation of selective autophagy.

## Conclusions

**Fig. 8:**
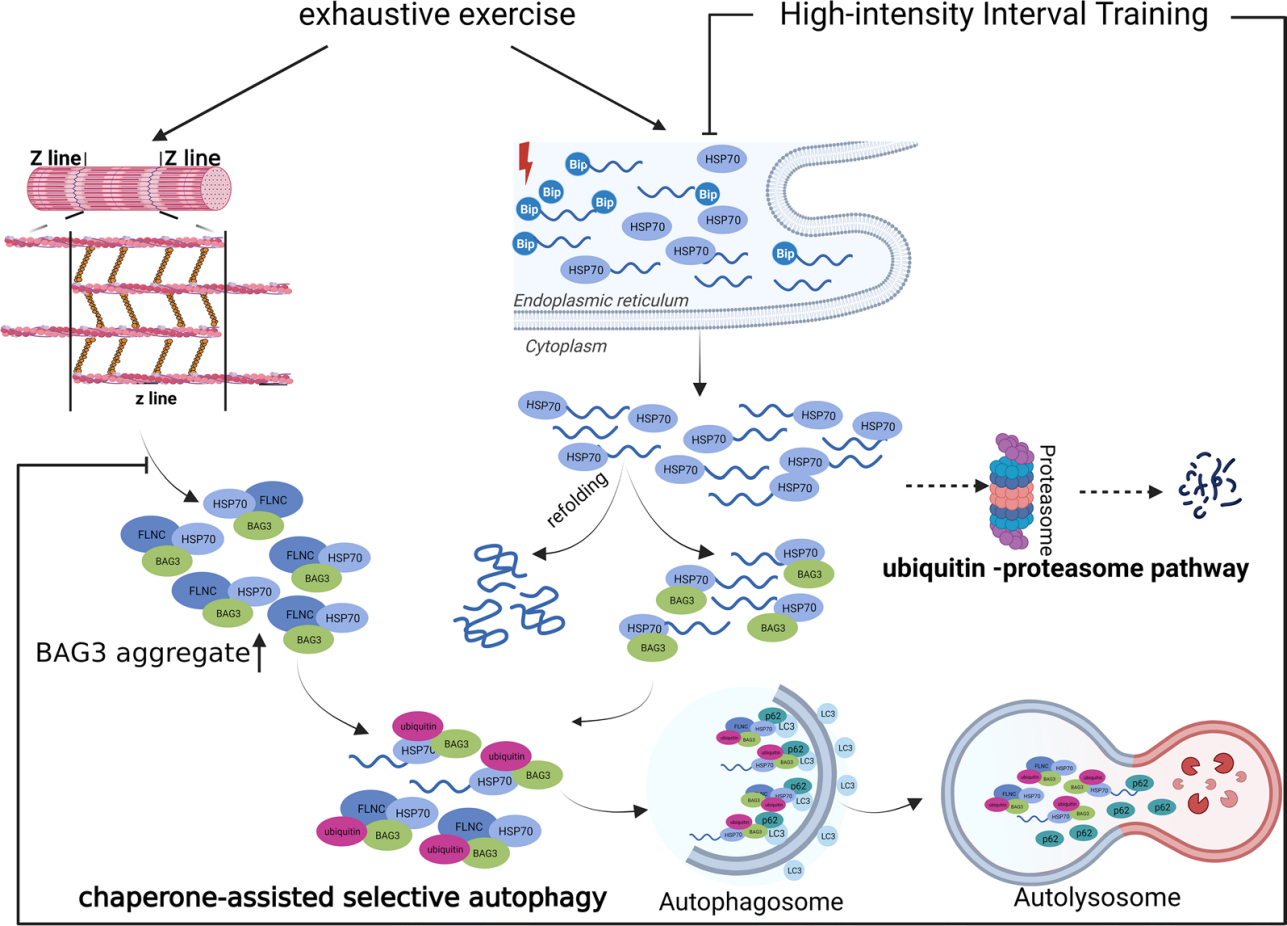
Improvement of myofibril damage and UPR induced by HIIT contribute to inhibiting the augmentation of selective autophagy in exhaustive exercise

In conclusion, we verify that p62-mediated selective autophagy was promoted during exhaustive exercise in response to the clearance of damaged, unfolded/misfolded proteins through assistance from the HSP70 chaperone complex, which might have a protective effect on maintaining the dynamic proteostasis against exhaustive exercise-induced muscle damage. Additionally, we also discovered pre-HIIT intervention decreased myofibril damage and UPR in subsequent exhaustive exercise, leading to a reduction of damaged, unfolded/misfolded proteins and an inhibition of CASA. Furthermore, with pre-HIIT intervention, overexpression of HSP 70 might facilitate refolding of damaged, unfolded/misfolded proteins caused by exhaustive exercise rather than degradation through CASA (Fig. 8).

### Supplementary Information

**Additional file 1: Figure S1.** Assessment of the degradation of autophagosomes in gastrocnemius muscle tissue. (A) Immunofluorescence staining of cathepsin D. Scale bars = 20 μm. (B) Analysis of immunoreactive area of cathepsin D (n=15). (C) Representative blots images of mature and intermediate cathepsin D and protein expression of cathepsin D (n=8). #P < 0.05 vs. Group C, *P < 0.05 vs. Group EE. Exhaustive exercise promotes mature cathepsin D expression and autophagosome degradation, pre-HIIT inhibits the degradation of autophagosomes. **Figure S2.** Assessment of exercise performance and muscle injury. (A) Running distance of rats until exhaustion on a treadmill; (B) alterations of plasma CK. Exhaustive exercise induces skeletal muscle injury, pre-HIIT ameliorated skeletal muscle injury and improved the exercise ability of rats.

## Data Availability

The data used to support the findings of this study are available from the corresponding author upon request.

## Abbreviations

CASA: Chaperone-assisted selective autophagy
HIIT: High-intensity interval training
BAG3: B-cell lymphoma 2-associated anthanogene
LC3: Microtubule-associated protein 1 light chain 3
UPR: Unfolded protein response
HSP: Heat shock protein
TEM: Transmission electron microscopy
LAMP-2: Lysosomal-associated membrane protein
