# Supplementary material for: High-intensity interval training alleviates exhaustive exercise-induced HSP70-assisted selective autophagy in skeletal muscle
Source: J Physiol Sci. 2023 Nov 21;73:32. doi: 10.1186/s12576-023-00884-2 (PMC10717669; doi:10.1186/s12576-023-00884-2)
Supplement: Supplementary file 1 — Additional file 1: Figure S1. Assessment of the degradation of autophagosomes in gastrocnemius muscle tissue. (A) Immunofluorescence staining of cathepsin D. Scale bars = 20 μm. (B) Analysis of immunoreactive area of cathepsin D (n=15). (C) Representative blots images of mature and intermediate cathepsin D and protein expression of cathepsin D (n=8). #P < 0.05 vs. Group C, *P < 0.05 vs. Group EE. Exhaustive exercise promotes mature cathepsin D expression and autophagosome degradation, pre-HIIT inhibits the degradation of autophagosomes. Figure S2. Assessment of exercise performance and muscle injury. (A) Running distance of rats until exhaustion on a treadmill; (B) alterations of plasma CK. Exhaustive exercise induces skeletal muscle injury, pre-HIIT ameliorated skeletal muscle injury and improved the exercise ability of rats. [file 12576_2023_884_MOESM1_ESM.pptx]

## Slide 1
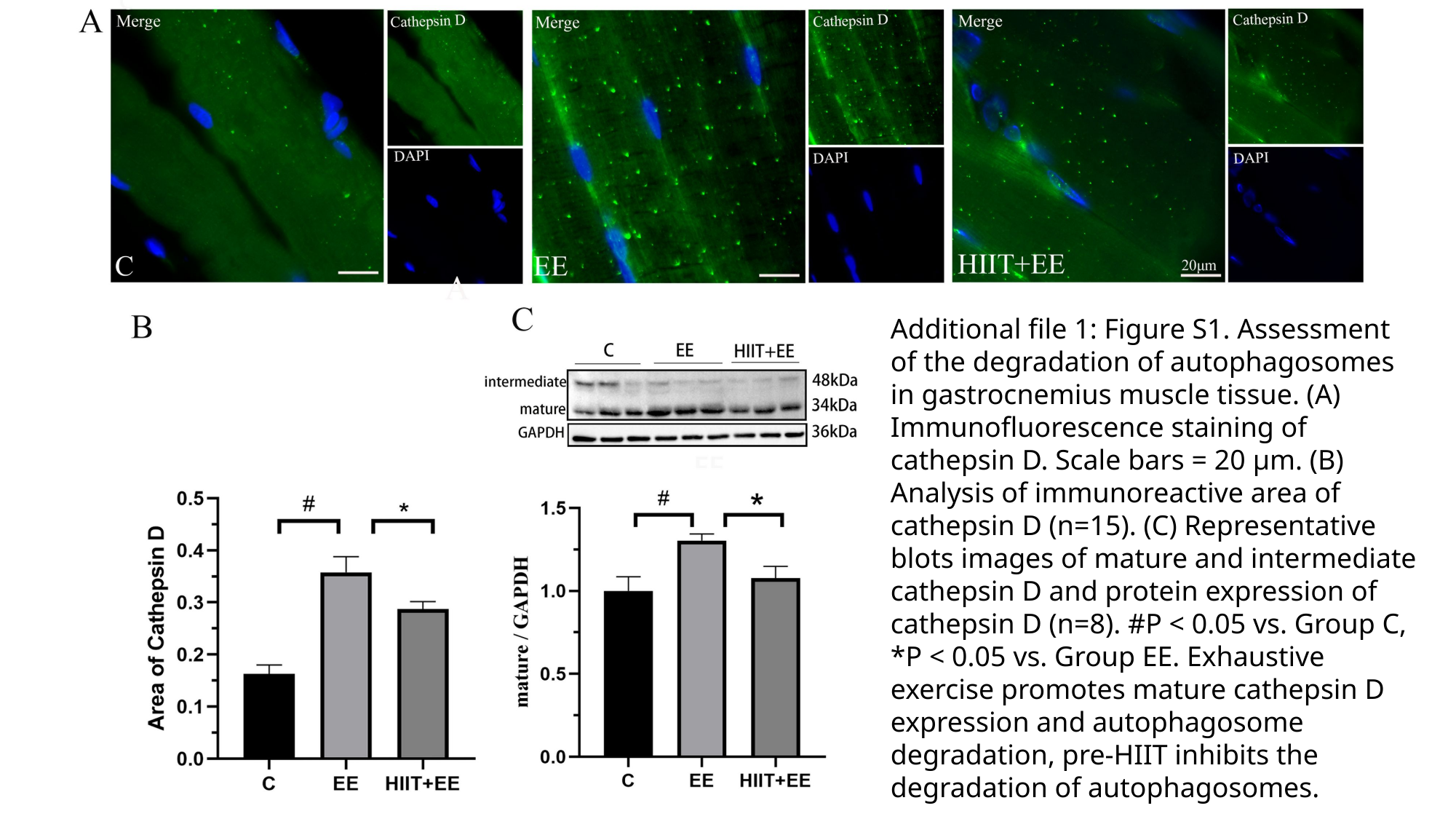

Additional file 1: Figure S1. Assessment of the degradation of autophagosomes in gastrocnemius muscle tissue. (A) Immunofluorescence staining of cathepsin D. Scale bars = 20 μm. (B) Analysis of immunoreactive area of cathepsin D (n=15). (C) Representative blots images of mature and intermediate cathepsin D and protein expression of cathepsin D (n=8). #P < 0.05 vs. Group C, *P < 0.05 vs. Group EE. Exhaustive exercise promotes mature cathepsin D expression and autophagosome degradation, pre-HIIT inhibits the degradation of autophagosomes.

## Slide 2
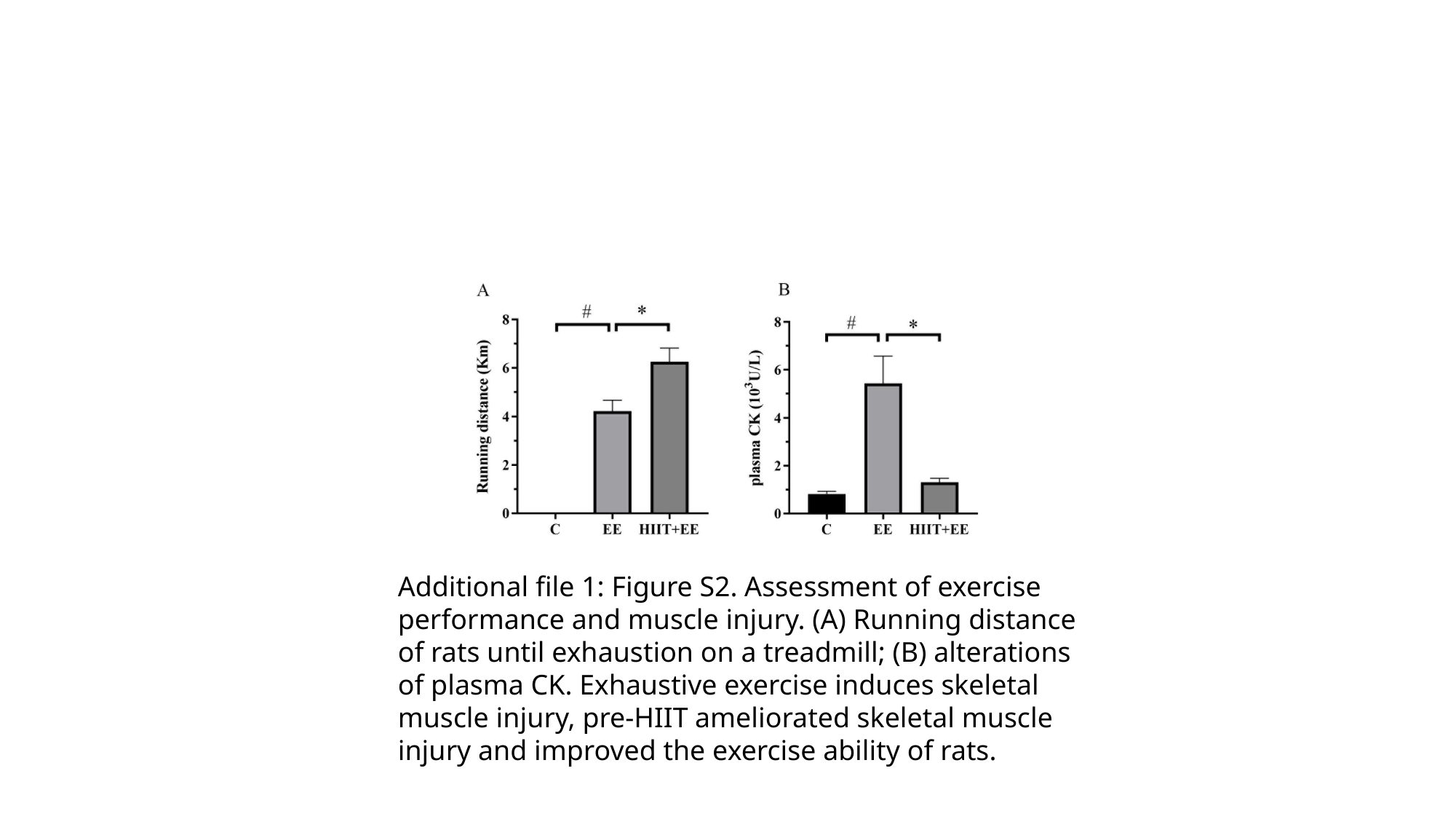

Additional file 1: Figure S2. Assessment of exercise performance and muscle injury. (A) Running distance of rats until exhaustion on a treadmill; (B) alterations of plasma CK. Exhaustive exercise induces skeletal muscle injury, pre-HIIT ameliorated skeletal muscle injury and improved the exercise ability of rats.
